# iProm-Sigma54: A CNN Base Prediction Tool for *σ*^54^ Promoters

**DOI:** 10.3390/cells12060829

**Published:** 2023-03-07

**Authors:** Muhammad Shujaat, Hoonjoo Kim, Hilal Tayara, Kil To Chong

**Affiliations:** 1 Department of Electronics and Information Engineering, Jeonbuk National University, Jeonju 54896, Republic of Korea; 2 School of Pharmacy, Jeonbuk National University, Jeonju 54896, Republic of Korea; 3 School of International Engineering and Science, Jeonbuk National University, Jeonju 54896, Republic of Korea; 4 Advanced Electronics and Information Research Center, Jeonbuk National University, Jeonju 54896, Republic of Korea

**Keywords:** bioinformatics, deep learning, computational biology, DNA promoters, convolutional neural networks, sigma factors

## Abstract

The sigma (σ) factor of RNA holoenzymes is essential for identifying and binding to promoter regions during gene transcription in prokaryotes. $\sigma^{54}$ promoters carried out various ancillary methods and environmentally responsive procedures; therefore, it is crucial to accurately identify $\sigma^{54}$ promoter sequences to comprehend the underlying process of gene regulation. Herein, we come up with a convolutional neural network (CNN) based prediction tool named “iProm-Sigma54” for the prediction of $\sigma^{54}$ promoters. The CNN consists of two one-dimensional convolutional layers, which are followed by max pooling layers and dropout layers. A one-hot encoding scheme was used to extract the input matrix. To determine the prediction performance of iProm-Sigma54, we employed four assessment metrics and five-fold cross-validation; performance was measured using a benchmark and test dataset. According to the findings of this comparison, iProm-Sigma54 outperformed existing methodologies for identifying $\sigma^{54}$ promoters. Additionally, a publicly accessible web server was constructed.

## 1. Introduction

The process of translating genetic data encoded in DNA into a matching RNA molecule is called transcription. RNA polymerase, an enzyme, reads the DNA code during transcription to create messenger RNA (mRNA), a single-stranded RNA molecule that retains the genetic material to the ribosome for protein synthesis. With the exception of the change of uracil for thymine in the RNA molecule, the mRNA molecule is a complimentary copy of the DNA sequence. Transcription is the initial step in gene expression and an important mechanism in regulating cellular activity [1]. A promoter is a DNA sequence that regulates transcription initiation throughout the transcription process. The promoter is the site on the DNA where the RNA polymerase enzyme attaches and begins the transcription process. The promoter sequence serves as a binding site for RNA polymerase and other regulatory proteins that govern transcription start, rate, and termination. The existence of unique promoter sequences enables gene expression regulation, allowing cells to react to various stimuli and govern the creation of certain RNA and protein products. Different kinds of promoters may result in varying degrees of gene expression and can decide whether a gene is tissue-specific or inducible. As a result, the promoter is an essential component in the control of gene expression. The sigma σ factor of RNA holoenzymes is essential for recognition and binding of promoter regions during gene transcription in prokaryotes [2]. As a result, prokaryotic promoters are classified corresponding to (σ) factors. Seven known σ factors exist, such as $\sigma^{19}$, $\sigma^{24}$, $\sigma^{28}$, $\sigma^{32}$, $\sigma^{38}$, $\sigma^{54}$, and, $\sigma^{70}$ and every (σ) factor serves a distinct purpose. The $\sigma^{54}$ factor can identify the consensus sequences TGCATTA and CTTGGCACTGA, which are around −12 base pairs (bp) and −24 bp upstream of the transcription start site (TSS), respectively. $\sigma^{54}$ promoters are involved in a variety of supporting activities and ecologically responsible practices [2], such as the extraction of chemotaxis transducers, construction of motility organs [3], nitrogen fixation [4], arginine catabolism [5], alginate biosynthesis [6], and flagella [7]. Therefore, understanding the procedure of gene transcription and establishing gene expression networks requires knowledge of $\sigma^{54}$ promoters. As a result, it is crucial to identify binding promoters for a specific σ factor for future research into gene regulation and functional genomics [8]. Since promoters are essential for gene transcription, precise identification of promoter locations has become essential for understanding gene expression, interpreting patterns, and building genetic regulatory networks. Several biological investigations have been carried out to determine promoters, including mutational analyses [9,10] and immunoprecipitation tests [11]. As the investigational discovery of promoters is costly and time taking, the computerized identification of σ factor promoters has emerged as an important topic in bioinformatics.

Promoter sequences may be given computationally as a classification problem, that is, as a way to identify whether a query sequence is a promoter of a particular σ factor, based on its feature characteristics. Promoter predictors may be divided into three types, namely the signal-based approach, the content-based approach, and the GpG-based approach, according to the methodology that was used to analyze them. The non-element parts of the sequence are disregarded by signal-based predictors in favor of the promoter elements that are directly connected to the RNA polymerase binding site. As a direct consequence of this, the accuracy of the forecast was poor and unsatisfying. The following are some examples of signal-based predictors: “PromoterScan” [12], which classified promoter sequences from non-promoter sequences using the extracted features of the TATA-box and a weighted matrix of transcription factor binding sites with a linear discriminator; “Promoter2.0” [13], which extracted the features from various boxes such as the TATA-Box, CAAT-Box, and GC-Box and then passed them to artificial neural networks. Ref. [14] utilized of TATA-Box and a classifier called a relevance vector machine (RVM). The content-based predictors rely on counting the frequency of k-mers by iteratively running a window of k-length length across the sequence. However, these approaches do not take into account the spatial information that is included in the sequences of base pairs. The following are some examples of content-based predictors: “PromFind” [15], which used the k-mer frequency to perform the hexamer promoter prediction; “PromoterInspector” [16], which recognized the regions containing promoters based on a common genomic context of polymerase II promoters by scanning for specific features defined as variable length motifs; and “MCPromoter1.1” [17]. In conclusion, GpG-based predictors made use of the position of GpG islands since the promoter region or the first exon region in human genes often includes GpG islands [18,19,20]. However, since only sixty percent of the promoters include GpG islands, the accuracy of predictions made using this form of predictor has never been higher than sixty percent. In recent years, techniques based on sequencing have been used in the process of promoter prediction. In order to accurately anticipate enhancer-promoter interactions [21] applied a variety of feature extraction algorithms. These tactics allowed them to obtain the most relevant sequence information. A sequence-based predictor, referred to as [22] proposed “PromoterPredict” in order to predict the strength of *Escherichia coli* promoters based on a dynamic multiple regression approach in which the sequences were represented as position weight matrices. This was done in order to determine whether or not a given promoter will be effective in activating transcription (PWM). In order to differentiate between promoter and non-promoter sequences, ref. [23] leveraged the variations in DNA sequence stability that exist between promoter and non-promoter sequences.

Machine Learning (ML) is an Artificial Intelligence (AI) application that allows autonomous learning and improvement from experience without explicit programming or knowledge of the learning environment. The primary objective is to design and build computational tools that can autonomously learn without human intervention. By definition, it must be able to adapt its behavior based on previous outcomes. ML has become one of the most important techniques for several genomics research objectives, including, the description and interpretation of huge genomic datasets, the Annotation of several sequence elements in genomics, and the prediction of genetic variation’s effect on DNA/RNA sequences. Diverse computational strategies for various research challenges have yielded excellent results in recent years [24,25,26,27]. A prediction based on sequences called “iPro70-PseZNC” [28] was developed to detect $\sigma^{70}$ promoters in prokaryotes. Samples of DNA sequences in the predictor were formed using a unique pseudo-nucleotide composition termed “PseZNC” which incorporates the six local DNA structural features and multi-window Z-curve composition. To extract the generic properties of bacterial promoters, a unique variable-window Z-curve approach was proposed to identify promoter sequences from two prokaryotic species: *Escherichia coli* and *Bacillus subtilis* [29]. A prediction tool, “iPromoter-FSEn” proposed a feature subspace-based ensemble classifier to extract features from DNA sequences and identify the $\sigma^{70}$ promoters [30]. To discover about $\sigma^{70}$ promoters in prokaryotes, “70ProPred” presented a position-specific trinucleotide propensity based on a single-stranded characteristic model [31]. An artificial neural network (ANN)-based tool, i.e., “SAPPHIRE” predicted $\sigma^{70}$ promoters in *Pseudomonas* sp. The sequence comparison to the −35 and −10 boxes of $\sigma^{70}$ promoters discovered experimentally in P. aeruginosa and P. putida is evaluated using “SAPPHIRE” [32]. A tool named “iPromoter-2L2.0” [33] proposed a two-stage model to identify promoter sequences and classify their type, in the first stage model predicts the given DNA sequence as a promoter and model the stage classifies the predicted promoter sequence as one of its class from $\sigma^{24}$, $\sigma^{28}$, $\sigma^{32}$, $\sigma^{38}$, $\sigma^{54}$, and $\sigma^{70}$. Kmer and PseKNC were used in “iPromoter-2L2.0” to extract discriminative features, while SVM was used as a classifier. “MULTiPly” [34], a new multilayer computational technique for predicting promoters and σ class from $\sigma^{24}$, $\sigma^{28}$, $\sigma^{32}$, $\sigma^{38}$, $\sigma^{54}$, and $\sigma^{70}$, was presented. “MULTiPly” considered both local information such as k-tuple nucleotide composition and dinucleotide-based autocovariance, as well as global information based on bi-profile Bayes and k-nearest neighbor feature extraction techniques. “iPromoter-BnCNN” [35] was presented for the accurate recognition and classification of six types of promoters: $\sigma^{24}$, $\sigma^{28}$, $\sigma^{32}$, $\sigma^{38}$, $\sigma^{54}$, and $\sigma^{70}$. It is a CNN-based model with three layers that uses parallel branching to incorporate local features related to the monomer nucleotide sequence: trimer nucleotide sequence, dimer structural features, and trimer structural attributes. “pcPromoter-CNN” [36] is a two-layer model that identifies the given sequence as a promoter or not a promoter and then classifies its σ type from $\sigma^{24}$, $\sigma^{28}$, $\sigma^{32}$, $\sigma^{38}$, $\sigma^{54}$, and $\sigma^{70}$. Recently a CNN-based tool “PromoterLCNN” [37] presented a two-layer model for bacterial promoter identification and classification from $\sigma^{28}$, $\sigma^{32}$, $\sigma^{38}$, $\sigma^{54}$, and $\sigma^{70}$.

Recently, computational methods for identifying and classifying sigma promoters have made great progress. However, we conclude that the current approaches need the following improvements:

- Most previous studies predicted promoter sequences from $\sigma^{70}$. Classifying the anticipated promoter sequences from $\sigma^{54}$ is uncommon.
- Not every study has produced a user-friendly and publicly accessible web server, making it difficult for most experimental scientists to use in practice.
- The false-positive predictions of the above-mentioned studies are remarkable because of the imbalanced dataset.
- The advancement of high-throughput whole-genome sequencing and the integration of verified promoter sequences has resulted in the development of databases such as *“Pro54DB”* [38], a database of $\sigma^{54}$ promoters. Therefore, there is a need for a computational model to identify $\sigma^{54}$ promoters, because databases play a vital role in development of computational tools.

**Figure 1:**
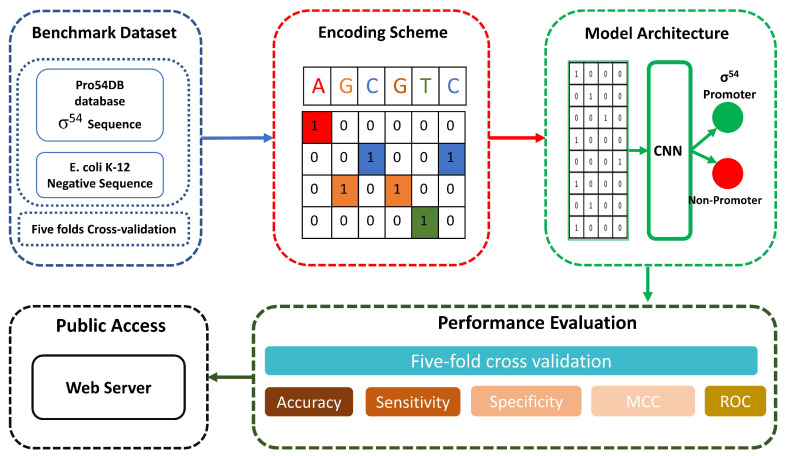
iProm-Sigma54 model flow diagram. We utilized $\sigma^{54}$ promoter sequences from the *“Pro54DB”* database and performed one-hot feature encoding scheme, after that we input encoded sequence to a optimal CNN model and later performed performance evaluation and finally made web server.

To overcome these limitations, we propose a CNN-based model “iProm-Sigma54” to identify $\sigma^{54}$ promoter sequences. First, we built a benchmark dataset and performed a one-hot feature-encoding scheme to extract the feature representation vector. We used a variety of assessment criteria that are often applied in bioinformatics to evaluate the model’s performance. The Matthews correlation coefficient (MCC) and the accuracy, sensitivity, and specificity were computed. To further examine the model, we used five-fold cross-validation assessment measures. Additionally, the receiver operating characteristic (ROC) curve was computed. And at last, a web server was created in accordance with the proposed model. Figure 1 shows the flow diagram of the proposed model.

## 2. Benchmark Dataset

For the creation and evaluation of computational approaches, an empirically validated benchmark dataset is crucial. This research utilized the recently updated $\sigma^{54}$ promoter sequences from the *“Pro54DB”* (i.e., $\sigma^{54}$ promoter database), that contains prokaryotic promoters that have been experimentally confirmed; *“Pro54DB”* was created to collect data on $\sigma^{54}$ promoters. The present edition includes 210 experimentally proven $\sigma^{54}$ promoters with 297 regulated genes in 43 species, which were collected from 133 research articles. Each positive sequence in the dataset is 81 bp long. A negative dataset of promoters utilized by “iPromoter-2L2.0”, “MULTiPly”, “iPromoter-BnCNN”, “pcPromoter-CNN”, and “PromoterLCNN” was used for the same. Furthermore, this study utilized CD-Hit [39] with the threshold value set to 0.8 to minimize duplication and reduce homologous bias. Consequently, the benchmark dataset *Ds* is mathematically expressed in Equation (1):

(1) $$Ds=Seq^{+}\cup Seq^{-}$$

where $Seq^{+}$ represents the $\sigma^{54}$ promoter sequences and $Seq^{-}$ depicts the negative sequences. We split the benchmark dataset *Ds* into further two sets called training and testing datasets and utilized 80% of the data for model training and five-fold cross-validation. Whereas 20% of the dataset was used to test the proposed model. Table 1 shows the parameters of the benchmark dataset.

## 3. Feature Encoding Scheme

A DNA sequence has four nucleotides (*A*, *T*, *C*, and *G*), and it must be translated into a numerical representation in order to execute computer operations. Thus, this study utilized a one-hot feature encoding scheme. Several recent cutting edge bioinformatics techniques have used this technique e [40,41,42,43]. Representation of each nucleotide for *A*, *C*, *G*, and *T*, characterized as follows:

A→(1,0,0,0)

C→(0,1,0,0)

T→(0,0,1,0)

G→(0,0,0,1)

Consequently, an (81,4) two-dimensional matrix can be used to represent each sample sequence.

## 4. Proposed Methodology

A 1D Convolutional Neural Network (1D-CNN) is a form of convolutional neural network used to handle one-dimensional signals such as time series data, audio signals, DNA/RNA sequence and text data expressed as word or character sequences. The convolutional layer of a 1D-CNN works on 1D filters rather than 2D filters as in standard CNNs for picture data. The filters move through the input sequence, calculating dot products between the filter weights and the local sequence components to generate a feature map that identifies certain patterns or features in the input sequence. 1D-CNNs, like standard CNNs, employ pooling layers to lower the spatial dimensions of the feature maps and generate more robust features. The pooling procedure, however, is performed along the time dimension of the feature maps rather than the spatial dimensions in a 1D-CNN. Finally, similar to standard CNNs, the extracted characteristics are input into one or more fully connected layers to create a prediction or classification. The 1D structure of the input data is used in these applications to efficiently learn meaningful representations and generate predictions. The most notable benefit of a CNN is that it does not need any previous feature information from data; instead, a model base on CNN can extract features directly from data. This study employed a CNN-based model to extract characteristics from a DNA sequence. CNN has demonstrated outstanding achievements in natural language processing, image processing [44,45] , and computational biology [46,47]. Several recent studies have performed hyperparameter tuning to improve the model for bioinformatics challenges. A grid search was performed on the training dataset to determine the optimal model, and six hyperparameters were tuned during the CNN learning process. Grid search is a hyperparameter tuning method used in AI to find the best set of hyperparameters for a model that optimize its performance on a given task. It is called grid search because it exhaustively searches over a specified hyperparameter grid, which is a predefined range of values for each hyperparameter. Table 2 lists the calibration ranges for each hyperparameter.

### CNN Architecture

After selecting the optimal model from the grid search, we performed a one-hot feature extraction technique on the dataset and input it to the CNN. The CNN consists of two one-dimensional convolutional layers (i.e., Conv1D), followed by max-pooling layers and dropout layers; The filter sizes of Conv1D are 128 and 258 whereas the kernel sizes of 5 and 7, respectively. The pooling size in both max-pooling layers was 4, with strides of 2. Following the second dropout layer, a flattened layer is used. Following this, we utilized a dense layer using 64 nodes, followed by a dropout layer. For all three dropout layers, the dropout value was 0.5. For all Conv1Ds and dense layers, we utilized the rectified linear unit (ReLU) activation function, following equation defines the ReLU:

(2) ReLU(p)=max(0,p)

The ReLU activation function is a simple threshold function used in neural networks, where negative values are set to zero and positive values are unchanged. This function is computationally efficient, introduces non-linearity, and is less prone to the vanishing gradient problem compared to other activation functions. We utilized a dense layer as an output layer with a single node and sigmoid activation function. The neuron’s output will always range from 0 to 1 when its activation function is a sigmoid function and based on that score classify the input sequence as a promoter or non-promoter. The following are mathematical ways to express the sigmoid function:

(3) $$Sigmoid\left(p\right)=\frac{1}{1+e^{-p}}$$

where *e* is the base of the natural logarithm and *p* is the input value. The function has an “S” shaped curve, which is why it is often referred to as the “sigmoid” function. To prevent the model overfitting, we employed bias regularization and L2 regularization with a value of 0.0001 in the convolution and dense layers. Bias regularization is a technique in machine learning that aims to prevent overfitting by penalizing the magnitude of the bias values in a model. L2 regularization, also known as weight decay, is a commonly used method for regularizing the weights in a model. It involves adding a penalty term to the loss function. The penalty term encourages the model to have smaller weights, which can prevent overfitting and lead to a more generalized model. We utilized the “Keras class weights” technique to handle the imbalanced data problem. The Keras framework was used to develop and train the “iProm-Sigma54” model, which uses binary cross-entropy as its loss function.

(4) $$H_{p}\left(q\right)=-\frac{1}{N_{pos}+N_{neg}}\left[\sum_{i=1}^{N_{pos}}\log\left(p\left(y_{i}\right)\right)+\sum_{i=1}^{N_{neg}}\log\left(1-p\left(y_{i}\right)\right)\right]$$

**Figure 2:**
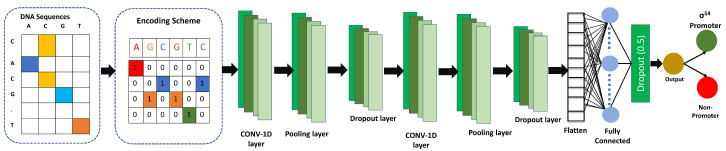
iProm-Sigma54 CNN Model Architecture. The CNN model consists of two one-dimensional convolutional layers, followed by max-pooling layers and dropout layers.

The binary cross-entropy function is given in Equation (4). Adam was utilized as an optimizer. There were 70 epochs in the batch, with a batch size of 32. Furthermore, an early stopping was imposed with a patience value of 30 to stop model training when the prediction accuracy doesn’t improve on the validation set. The proposed CNN model’s design is shown in Figure 2.

## 5. Results and Discussion

### 5.1. Evaluation Metrics

Five-fold cross-validation was used to assess the proposed model’s classification performance. This study employed four distinct measures to evaluate the effectiveness of “iProm-Sigma54”, which has previously been used in other state-of-the-art approaches. The specificity (*Sp*), sensitivity (*Sn*), accuracy (*Acc*), and *MCC* were the measurements. *Sp* computes the percentage of true negatives among all negatives (false positive rate), i.e., how well the model accurately recognizes negative cases. Whereas *Sn* measures the percentage of true positives among all positives (true positive rate), i.e., how well the model correctly identify positive cases and *Acc* measures the overall percentage of correct calculations (both true positive and true negative) among all cases. *MCC* is a measure that takes into account true and false positives and negatives and provides a balanced measure of the accuracy of binary classifications. It ranges from −1 to 1, with 1 indicating perfect prediction, 0 indicating random prediction, and −1 indicating perfect inverse prediction. The following are the mathematical expressions for these metrics

(5) $$Sp=\frac{PN}{PN+PP}$$

(6) $$Sn=\frac{PP}{PP+FN}$$

(7) $$Acc=\frac{PP+PN}{PP+PN+FP+FN}$$

(8) $$MCC=\frac{PP\times PN-FP\times FN}{\sqrt{\left(PP+FP)(PP+FN)(PN+FP)(PN+FN\right)}}$$

In the above equations from Equations (5)–(8), *PP* denotes the number of true positives whereas, *PN* stands for true negatives, *FP* and *FN* denote the number of false positives, and false negatives, respectively.

### 5.2. Results and Comparison

**Figure 3:**
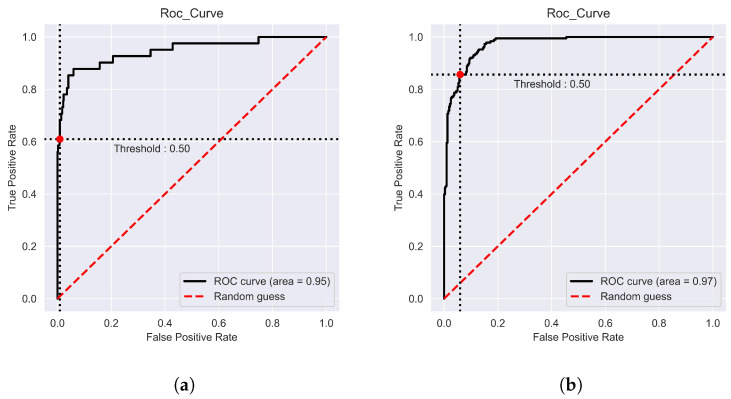
Receiver Operating Characteristic (ROC) curves The AUC ranges from 0.5 (for a random classifier) to 1 (for a perfect classifier): (**a**): ROC Curve on 5 fold (**b**): ROC Curve on Test Dataset.

Using five-fold cross-validation, the “iProm-Sigma54” prediction performance was assessed. The parameters utilized in this study were the same as those used to choose the best model, and ROC curves were also considered. During cross-validation, iProm-Sigma54 obtained an *Acc* of 95.45%, *Sn* of 96.53%, *Sp* of 90.64%, *MCC* of 0.848, and AUROC of 0.95; suggesting that the proposed predictor is capable of properly recognizing whether a query sequence is of the $\sigma^{54}$ promoter. Similar performance measures were performed using “iPro54-PseKNC”, “iPromoter-2L”, “iPromoter-BnCNN”, and “PromoterLCNN”, which are considered cutting-edge approaches for diagnosing and classifying σ promoters. Compared to results obtained using state-of-the-art techniques, “iProm-Sigma54” exhibited improved performance. A noticeable improvement by “iProm-Sigma54”, in terms of sensitivity and specificity, assures that the proposed model reduces the false positive values. A notable improvement of 5.8% in the value of *MCC* is evidenced that the proposed method accurately distinguishing between the promoter and non-promoter classes. Table 3 shows the iProm-Sigma54 performance and the comparison results with state-of-the-art methods. Figure 3a depicts the ROC curve for predicting the $\sigma^{54}$ promoter on the cross validation dataset; the curve evidently indicates a significant area under the curve.

A test dataset was used to determine the authenticity of iProm-Sigma54. it achieved an *Acc* of 98.40%, *Sn* of 95.12%, *Sp* of 97.19%, *MCC* of 0.9113 and AUROC of 0.97 on the test dataset. Table 4 shows the comparison results on test dataset. Figure 3b illustrate ROC curve for test dataset; the curve evidently indicates a significant area under the curve

**Figure 4:**
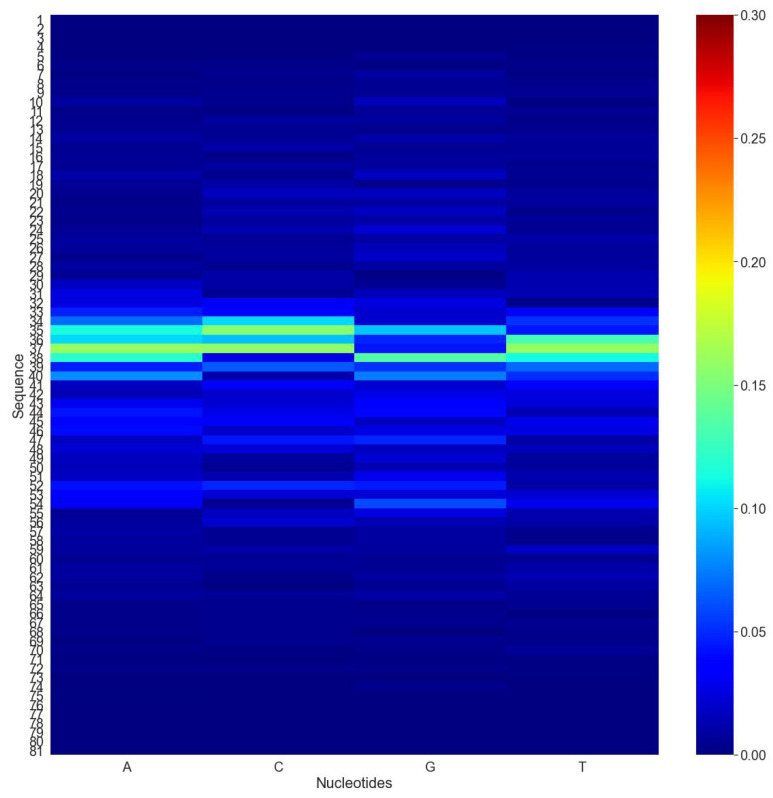
Computational mutation scanning was conducted for each promoter sequence in the test set to determine whether changing each base of the input subsequence affected. Red indicates a high frequency of mutations, while blue indicates a low frequency of mutations.

For a more thorough examination, we investigated the effect of alternating nucleotides at each location on the performance score. Computational mutation scanning was conducted for each promoter sequence in the test set to determine whether changing each base of the input subsequence affected the results. Sequences with a length of 81 bp were considered and nucleotides at any position in the sequence were mutated individually. In addition, the absolute differences between the original and mutated sequence predictions were calculated and stored. The average expected score for all mutations was determined for all the sequences. Finally, the heat map shown in Figure 4 was generated using the average predicted score. As seen in the heat map, mutations in the middle of the sequence have a greater impact on the “iProm-Sigma54” CNN estimation than mutations in the beginning and ending nucleotides of the sequence, respectively. The results revealed that nucleotide changes had little impact on the outcome of “iProm-Sigma54” CNN recognition.

**Figure 5:**
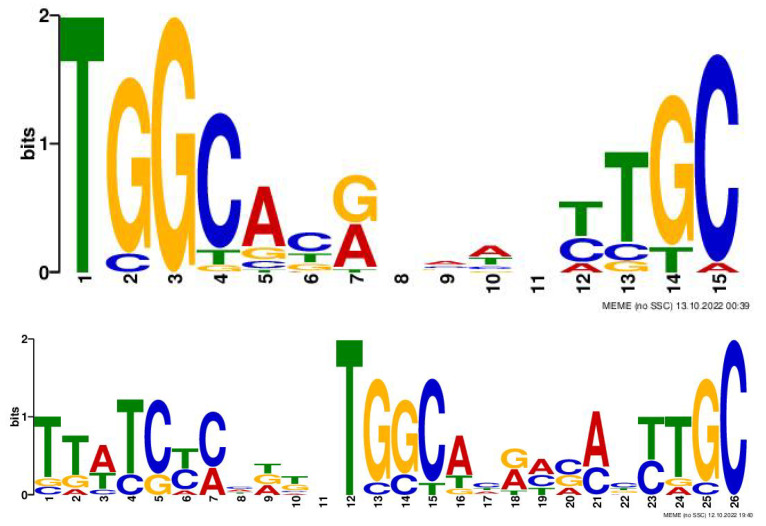
Motif Analysis: Learned motifs of the kernels of the first Conv1D can be considered position weight matrices (PWMs).

The learned motifs of the kernels of the first Conv1D can be considered position weight matrices (PWMs). Visualization of motifs offers a biologist further insight into the promoter regions. Figure 5 shows the instances of sequence motifs found using the proposed methodology.

## 6. Webserver

To make the suggested tool more accessible to the scientific community, The high-performance “iProm-Sigma54” tool is hosted on a web server that is accessible at http://nsclbio.jbnu.ac.kr/tools/iProm-Sigma54/. This approach has been used by several scholars [48,49,50]. Researchers and experts in medicine and bioinformatics can use “iProm-Sigma54” a simple-to-use tool. The guidelines to use the webserver are as follows:

- It accepts input using two different methods: direct sequence input and file uploading with sequences with up to one thousand sequences in a FASTA format. To upload a file format must be “.fa”.
- Set the threshold value ranges from 0–1.
- Click on “submit Sequence” button for the prediction results

**Figure 6:**
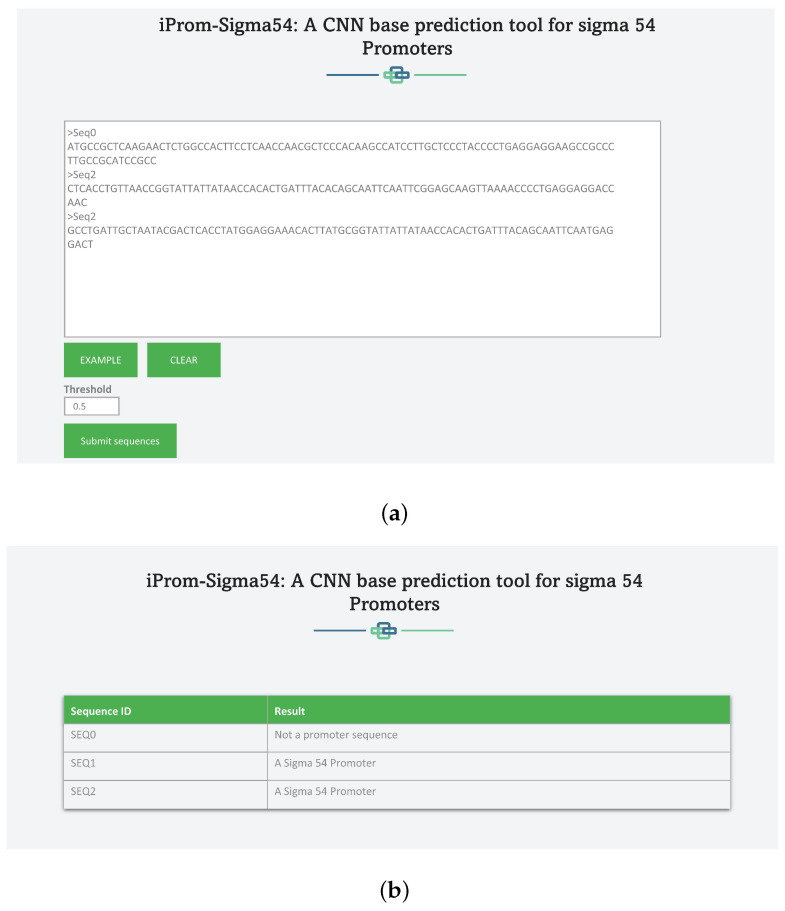
Webserver of iProm-Sigma54: (**a**) Adding Sequences for Prediction to Web server. The Query Sequence should be in Fasta format and must contain only *A*, *C*, *G*, *T*. (**b**): Predictor Output for Query Sequences. Table shows output for each query sequence.

A web server snippet is shown in Figure 6, where Figure 6a displays writing query sequences whereas the Figure 6b shows the prediction results.

## 7. Conclusions

The study of the $\sigma^{54}$ promoter and its role in gene regulation is an important area of research in the field of bioinformatics, as it can provide insights into the mechanisms by which bacteria respond to their environment and the ways in which they are able to adapt to changing conditions. This information can be used to develop new strategies for controlling bacterial populations and for preventing the spread of bacterial infections. Therefore, precise identification of $\sigma^{54}$ promoter sequences is essential for understanding the underlying mechanisms of gene regulation. This study established the “iProm-Sigma54” model for this effect, which is based on a convolution neural network; a grid search algorithm was used to build a CNN-based predictor. A one-hot encoding scheme was used to generate the input matrix for the CNN. This study used five-fold cross-validation training and evaluated the results using a test dataset. The proposed method outperforms the most competitive methods in the literature. Motif and heatmap analyses were performed to provide more biological insights into the model. Finally, we made an online web server that would give convenience to other experimental scientists. In addition, this study provides a training model and dataset at GitHub: github.com/Shujaatmalik/iProm-Sigma54.

## Figures and Tables

**Table 1:** Parameters of benchmark and test dataset.

Class	Benchmark Dataset	Test Dataset	Sequence Length
$\sigma^{54}$ Promoter	168	42	81 bp
Non-Promoter	2288	500	81 bp

**Table 2:** Ranges of Parameters in Hyperparameter tuning.

Parameters	Range
Number of Conv1D	[2, 3, 4, 5]
Filters Size in Conv1D	[8, 12, 16, 22, 32, 42, 64, 128]
Kernel Sizes in Conv1D	[2, 3, 4, 5, 6, 7, 8, 10, 12, 14]
Max-pooling Pool Size	[2, 4, 6]
Max-pooling Stride length	[2, 4]
Values of Dropout	[0.2, 0.25, 0.3, 0.35, 0.4, 0.45, 0.5]
Neurons of Dense Layer	[8, 16, 32, 64, 80, 100]

**Table 3:** $\sigma^{54}$ Promoter and non-promoter identification comparison using 5-Fold on benchmark.

Model	*Acc* (%)	*Sn* (%)	*Sp* (%)	*MCC*
iPromoter-2L	94.04	53.19	99.57	0.65
iPro54-PseKNC	78.57	86.96	70.19	0.58
iPromoter-BnCNN	99.3	74.4	99.8	0.78
PromoterLCNN	99.4	68.0	99.9	0.80
iProm-Sigma54	95.45	96.53	90.64	0.858

**Table 4:** $\sigma^{54}$ Promoter and non-promoter identification comparison on a test dataset.

Model	*Acc* (%)	*Sn* (%)	*Sp* (%)	*MCC*
iPromoter-2L	81.23	92.27	63.57	0.483
iPro54-PseKNC	78.52	97.56	76.95	0.436
iPromoter-BnCNN	92.98	57.14	95.23	0.516
iProm-Sigma54	98.40	95.12	97.19	0.9113

## Data Availability

This study provides a training model and dataset at GitHub: github.com/ Shujaatmalik/iProm-Sigma54.
